# Low Cell Bioenergetic Metabolism Characterizes Chronic Lymphocytic Leukemia Patients with Unfavorable Genetic Factors and with a Better Response to BTK Inhibition

**DOI:** 10.3390/cimb46060305

**Published:** 2024-05-22

**Authors:** Simone Mirabilii, Monica Piedimonte, Esmeralda Conte, Daniele Mirabilii, Francesca Maria Rossi, Riccardo Bomben, Antonella Zucchetto, Valter Gattei, Agostino Tafuri, Maria Rosaria Ricciardi

**Affiliations:** 1 Hematology, Department of Clinical and Molecular Medicine, Sant’Andrea University Hospital, Sapienza University of Rome, 00189 Rome, Italy; mpiedimonte@ospedalesantandrea.it (M.P.); econte@ospedalesantandrea.it (E.C.); agostino.tafuri@uniroma1.it (A.T.); 2 WS Audiology, 91058 Erlangen, Germany; daniele.mirabilii@wsa.com; 3 Clinical and Experimental Onco-Hematology Unit, Centro di Riferimento Oncologico, I.R.C.C.S., 33081 Aviano, Italy; frossi@cro.it (F.M.R.); rbomben@cro.it (R.B.); zucchetto.soecs@cro.it (A.Z.); vgattei@cro.it (V.G.)

**Keywords:** bioenergetic metabolism, Chronic Lymphocytic Leukemia, BTK inhibition

## Abstract

Chronic Lymphocytic Leukemia (CLL) is an indolent malignancy characterized by the accumulation of quiescent mature B cells. However, these cells are transcriptionally and translationally active, implicating an active metabolism. The recent literature suggests that CLL cells have an oxidative-type phenotype. Given the role of cell metabolism, which is able to influence the outcome of treatments, in other neoplasms, we aimed to assess its prognostic role in CLL patients by determining the ex vivo bioenergetic metabolic profile of CLL cells, evaluating the correlation with the patient clinical/biological characteristics and the in vivo response to BTK inhibitor treatment. Clustering analysis of primary samples identified two groups, characterized by low (CLL low) or high (CLL high) bioenergetic metabolic rates. Compared to the CLL high, CLL with lower bioenergetic metabolic rates belonged to patients characterized by a statistically significant higher white blood cell count and by unfavorable molecular genetics. More importantly, patients in the CLL low cluster displayed a better and more durable response to the BTK inhibitor ibrutinib, thus defining a bioenergetic metabolic subgroup that can benefit the most from this therapy.

## 1. Introduction

Chronic Lymphocytic Leukemia (CLL) is an indolent malignancy characterized by the accumulation of quiescent, immunologically dysfunctional mature B cells which are CD5 positive and that fail to undergo apoptosis, leading to leukocytosis and absolute lymphocytosis, lymphadenomegaly, and splenomegaly. CLL cells reside in bone marrow, spleen, lymph nodes, and peripheral blood, being exposed to different microenvironments [1,2,3]. CLL cells, even if known as slowly proliferating or quiescent, are transcriptionally and translationally active, suggesting an active metabolism [4,5]. Antigenic stimulation, and, therefore, the constitutive activation of B-cell receptor (BCR) signaling plays a fundamental role in CLL pathogenesis by supporting the growth and survival of CLL cells [4,6,7]. Thus, the signaling pathways activated by this receptor represent elective targets for molecular therapies.

Aberrant signaling by the activation of BCR has a profound impact on CLL cell metabolism [5]. According to recent data, CLL cells have an increased oxidative phosphorylation despite a glucose consumption comparable to their healthy counterpart [8,9,10]. Although a certain degree of activity is present at the level of the glycolytic pathway, this does not seem to play a fundamental role in the metabolism of CLL, unlike other lymphoproliferative neoplasms characterized by a more pronounced proliferation [8,11]. Moreover, the transformation of CLL in Richter’s syndrome has been reported to be associated with an increased uptake of FluoroDeoxyGlucose (FDG) [12]. *ATM* and *TP53*, in addition to their role in controlling genomic integrity, also appear to be central regulators of carbon metabolism, thus correlating with metabolic and molecular profile [13]. In fact, del(11q) CLL cells would appear to be particularly sensitive to inhibition of glycolysis [14]. Similarly, Kluckova K et al. [15] reported that CLL cells in proliferation centers of several samples from patients with deletion of chromosome 17p (del17p) manifested increased spontaneous aerobic glycolysis in the BCR-unstimulated state (suggestive of a BCR-independent metabolic phenotype). The *TP53* mutation or del17p seem to confer a greater glucose dependence to these cells. *TP53* in fact limits glucose uptake by repression of GLUT1 and GLUT4, and regulates glycolysis by activating the transcription of TIGAR (TP53-inducible regulator of glycolysis and apoptosis) [16,17]. TIGAR is expressed by primary CLL cells, and hijacks the use of glucose-6-phosphate on the pentose phosphate (PPP) pathway [16,18]. The overexpression of oxoglutarate dehydrogenase and isocitrate dehydrogenase, two enzymes that facilitate both the oxidation and reduction in alpha-ketoglutarate, supports the hypothesis that these lymphocytes are characterized by a sustained oxidative phosphorylation [9].

Furthermore, CLL cells have an increased number in mitochondria and an increased production in ROS compared to normal B cells [11]. Other studies have shown that fatty acid metabolism plays an important role in leukemogenesis, including CLL [9]. It has been shown that CPT1 is overexpressed in CLL cells, and its inhibition by etomoxir causes cytotoxic effects on the cells, accompanied by an increase in ROS levels [13,19,20]. CPT1, therefore, represents a promising therapeutic target in CLL [21]. Previously, our group has shown that the use of a CPT1 inhibitor, ST1326, in AML, ALL, and CLL cells is able to determine cell growth arrest, mitochondrial damage, and the induction of apoptosis [21]. Taken together, these data indicate an active metabolism to CLL cells. Given the role of metabolism in other neoplasms that are able to influence the outcome of treatments, we aim to further assess its prognostic significance in CLL. To this end, we have determined the ex vivo cellular bioenergetic metabolic profile of CLL patients, both untreated and with a history of prior treatment, evaluating the correlation between cellular bioenergetic signature, clinical/biological characteristics, and response to BTK inhibitor treatment.

## 2. Materials and Methods

### 2.1. CLL Patients

Primary samples from CLL patients (*n* = 35) were collected after written informed consent from subjects admitted for diagnostic and therapeutic procedures to the Hematology of the University Hospital Sant’Andrea-Sapienza, Rome, Italy. The overall population included untreated patients and patients with a history of prior treatments. Clinical characteristics and treatment history for each patient are reported in Supplementary Table S1.

After sampling for bioenergetic metabolic profile, 13 of the 35 patients included in the study underwent ibrutinib treatment at different times, according to clinical and prognostic genetic criteria. All patients received the standard dose of 420 mg/die of ibrutinib. Treatment response was defined according to the criteria proposed by iwCLL [22]. The follow-up was at least monthly.

### 2.2. CLL Cell Enrichment

Primary samples from CLL patients (percentage of CD19+ cells ranged from more than 81% to 87% according to flow cytometry in lower and higher WBC count, respectively) were enriched in CLL cells using Lympholyte-H (Cederlane, Burlington, ON, Canada) density-gradient centrifugation, counted, and immediately used for metabolic measurements, as described below. 

### 2.3. B-Cells Purification

Peripheral blood normal B cells (*n* = 6) were purified from buffy coats using Ficoll-Hypaque (GE Healthcare, Chicago, IL, USA) density gradient centrifugation, followed by magnetic-activated cell sorting to select CD19+ cells (CD19 Microbeads; Miltenyi Biotec, Bergisch Gladbach, Germany, GER). B cell purity, as analyzed using flow cytometry for CD20+, was >90%.

### 2.4. Flow Cytometry

CD49d expression was analyzed using 3-color immunofluorescence by combining anti-CD49d-PE (clone 9F10), anti-CD5-FITC, and anti-CD19-PerCP-Cy5.5 or anti-CD19-APC (all from BD Biosciences, Franklin Lakes, NJ, USA). CD49d expression data were reported as the percent of CD5+CD19+ CLL cells displaying specific fluorescence intensity greater than the 98% to 99% of the same unstained population. The cutoff of 30% of CD49d expressing cells was employed to define CD49dhigh cases, as previously reported [23].

### 2.5. Cytogenetic and Molecular Analysis

Interphase FISH was performed to detect del17p, 11q22.3 deletion (del11q), 13q14 deletion (del13p), and trisomy 12 (tris12), as reported in [24].

Mutational status of *TP53*, *NOTCH1*, *SF3B1*, *BIRC3*, *C481S* (BTK gene), *R665W*, *L845F*, and *S707Y* (PLCG2 gene) related to ibrutinib resistance was assessed using next generation sequencing (NGS), with an amplicon-based strategy with at least 2000x coverage covering exons 2 to 11 for *TP53*, the whole exon 34 and part of 3′ untranslated region for *NOTCH1*, exons 12 to 17 for *SF3B1*, and exons 6 to 9 for *BIRC3*.

Sequencing analysis of *IGHV* was performed on either genomic DNA or complementary DNA using consensus primers for the *IGHV* leader or the *IGHV* FR1 regions in conjunction with JH primers, according to LymphoTrack *IGHV* Leader or *IGHV* FR1 assays (Invivoscribe, Shanghai, China). Sequences were analyzed using the IMGT databases and the IMGT/V-QUEST tool (http://imgt.org/, version 3.2.17, accessed on 2 April 2020).

### 2.6. Bioenergetic Metabolic Measurements

Bioenergetic metabolic rates were measured using a Seahorse XFp Extracellular Flux Analyzer (Agilent Technologies, Santa Clara, CA, USA), as previously published [25]. Briefly, 5 × 10^5^ CLL cells/well were resuspended in unbuffered DMEM medium, supplemented with 2 mM L-glutamine, 11 mM glucose and 1.2 mM pyruvate, and adjusted to pH 7.35. CLL cells were transferred in the XFp-dedicated plates pre-coated with poly-L-lysine (Merck KGaA, Darmstadt, Germany), centrifuged for 5 min at 1000 rpm with no acceleration and no brakes, and then incubated for 30 min at 37 °C in a CO_2_-free incubator. Oxygen Consumption Rates (OCR) and ExtraCellular Acidification Rates (ECAR) were measured for the basal state and following the sequential injection of oligomycin (1 μM), carbonyl cyanide-4-(trifluoromethoxy) phenylhydrazone (FCCP) (0.4 μM), and a mix of antimycin A (2 μM) and rotenone (2 μM) (all reagents from Merck KGaA, Germany) in each well, according to Seahorse Mito Stress Test protocol [26]. The data of the experiments were analyzed through dedicated software (XF Wave version 2.4.0, Agilent Technologies, CA, USA). Mito stress test parameters were been calculated according to the manufacturer’s instructions. OCR and ECAR values from the third measurement point, corresponding to the basal state, before any injection, were used for the following clustering analyses.

### 2.7. Statistics

Clustering analyses on patients were performed using Matlab 2018b (Mathworks, Natick, MA, USA) and the File Exchange function (Bart Finkston. MATLAB Central File Exchange, https://www.mathworks.com/matlabcentral/fileexchange/10161-mean-shift-clustering, 2021), aiming at segregating the population into subgroups. Results were analyzed through different clustering approaches to ensure a statistically correct group definition. In this respect, finding the optimal number of clusters given in a population is a well-known challenge, as most clustering methods require an a priori guess. The Mean-Shift Clustering (MSC) [27] was selected, since it does not require prior knowledge of the number of clusters. The MSC can be tuned using a single parameter, i.e., the window radius. The latter is the width of the search area where the mean of the selected datapoints is computed, yielding a centroid which is then updated iteratively. All the points lying within the radius distance from the centroid are then labelled as belonging to one cluster. The clustering was performed in three steps: (1) we obtained a candidate number of clusters (*n*) by running the MSC 48 times using an increasing radius at each run, (2) we selected the number of clusters *(n*) that occurred the most among all the runs, and (3) we used the selected number of clusters to segregate the population into subgroups. In the first step, we computed the Euclidean distance between all data points to identify the test range of the radius which was set within the minimum and the mean distance. In the third step, the radius was selected as the mean of all the radius values which yielded the most occurring number of clusters.

Student’s T test has been applied with *p* < 0.05 as the level of significance, using Microsoft Excel 2010 (Microsoft, Redmond, WA, USA) for mitochondrial parameter analysis. For enrichment analysis on clinical and biological patient characteristics, a one way-χ^2^ has been applied with *p* < 0.05 as level of significance (MedCalc Software Ltd. One-way Chi-squared test version 22.023: https://www.medcalc.org/calc/chisquared-1way.php, accessed on 29 April 2024).

## 3. Results

### 3.1. Bioenergetic Metabolic Clustering

**Figure 1:**
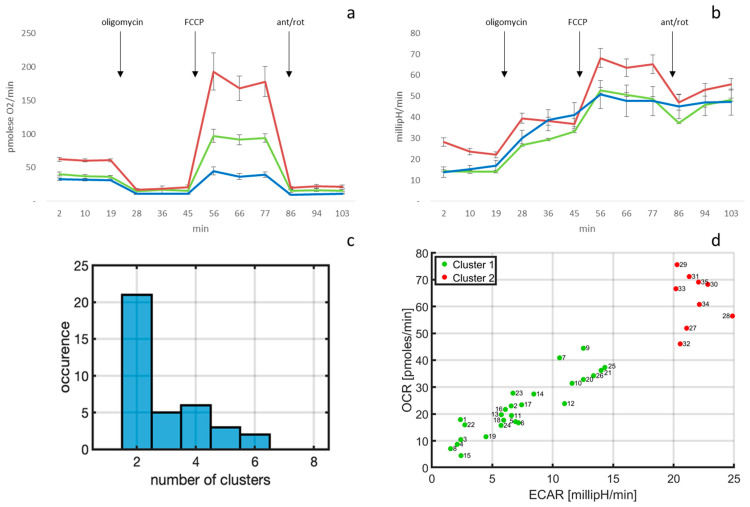
Clustering of CLL patients’ B-cells using rates of basal ECAR and OCR. (**a**) Example of a mito stress test analysis (see Section 2.6) for a CLL high patient (n. 34, red), CLL low patient (n. 21, green), and a normal b cell (blue) (ant/rot: antimycin A and rotenone). (**a**) Shows OCR curves, while (**b**) shows ECAR curves, obtained at the same time from the same samples. ECAR and OCR values corresponding to the third measurement points (min. 19) for 35 CLL patients have been used for the clustering analysis (**c**). (**c**) Number of clusters histogram obtained with 48 runs of the MSC: two clusters is the most frequent result. (**d**) Scatter plot of the patient distribution based on ECAR and OCR values, where each color corresponds to a cluster obtained with MSC and r = 17.5. Red indicates the CLL high patients’ cluster, whereas green indicates the CLL low patients’ cluster.

Cells from 35 CLL patients (Table 1) have been analyzed for basal rates of ECAR and OCR and compared to normal B cells obtained and purified from 6 healthy donors (Figure 1a,b). We used the third measurement points as reported in Section 2.6. We applied the MSC to cluster this data using the procedure described in Section 2.5. The occurrences of the number of clusters *n* among 48 runs of the MSC are shown in Figure 1c, where the value *n* = 2 was the most frequent, and was therefore selected to identify the radius value r = 17.5. A realization of the MSC with r = 17.5 is shown in Figure 1d.

The clustering analysis showed that the 35 analyzed patients could be segregated into two different clusters, defined as CLL high bioenergetic metabolism (CLL high) and CLL low bioenergetic metabolism (CLL low) (Figure 1d and Table 1).

The CLL low cluster was composed of 26 patients that showed a bioenergetic metabolic phenotype characterized by low levels of ECAR (mean value 7.35 ± 4.06 millipH/min) and OCR (mean value 22.59 ± 10.95 pmolesO_2_/min) compared to the remaining 9 patients, and CLL high, which displayed a significantly higher bioenergetic metabolic phenotype both in terms of ECAR (mean value 21.70 ± 3.39 millipH/min, *p* < 0.005) and of OCR (mean value 62.87 ± 11.70 pmolesO_2_/min, *p* < 0.005) (Table 1).

Moreover, CLL low cells showed an oxidative metabolism like normal B cells (OCR mean value 22.59 ± 10.95 vs. 25.87 ± 12.86 pmolesO_2_/min, respectively) and a significantly lower ECAR (mean value 7.35 ± 4.06 vs. 14.08 ± 7.93 millipH/min, respectively, *p* = 0.002).

**Figure 2:**
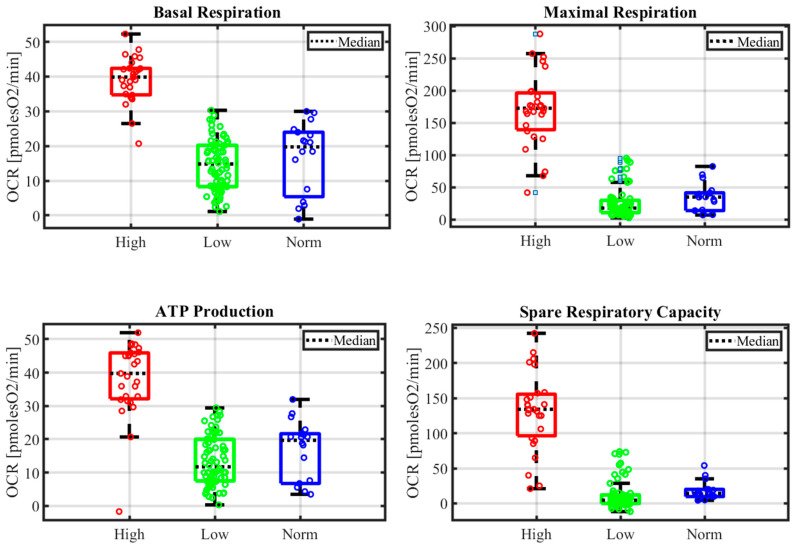
Boxplots of mito stress test parameters (see Section 2.6) in CLL high (High), CLL low (Low), and in normal B cells (Norm). The whiskers extend from the nonoutlier minimum to the lower quartile and from the upper quartile to the nonoutlier maximum.

Mitochondrial metabolism has been further dissected by analyzing the parameter derived from the Mito stress test protocol (see Section 2.6). In total, 34 out of 35 patients were evaluated (Supplementary Table S2). The results showed differences between the two CLL groups in terms of basal respiration (39.00 ± 6.62 vs. 14.45 ± 7.25 pmolesO_2_/min, in CLL high and CLL low, respectively, *p* < 0.005), maximal respiration (169.82 ± 57.64 vs. 26.19 ± 24.08 pmolesO_2_/min, *p* < 0.005), spare respiratory capacity (130.82 ± 54.98 vs. 11.73 ± 20.50 pmolesO_2_/min, *p* < 0.005), and ATP production (39.63 ± 11.14 vs. 13.57± 7.51 pmolesO_2_/min, *p* < 0.005) (Figure 2). Other parameters (proton leak and spare respiratory capacity as the percentage and coupling efficiency) showed no significant differences.

### 3.2. Clinical/Biological Correlations

When analyzed, according to a clinical–biological perspective, the two patient clusters that were characterized by a statistically significant different White Blood Cell Count (WBC): CLL low patients had a median of 48,200 (range 15,300–159,000), compared to a median of 15,100 (range 3880–32,290) in CLL high patients (*p* = 0.002).

According to cytogenetic and molecular characteristics (Table 2), we found that the CLL low cluster were enriched in patients bearing del(13q) (10 out of 26 CLL low patients) vs. only one case in CLL high. In contrast, three out of eight CLL high patients were characterized with trisomy of chromosome 12 vs. one patient in CLL low, all with high CD49d expression. All patients carrying the del(11q) and the multiple deletions, three del(13q);del(11q) and three del(13q);del(17p) are in the CLL low group. Similarly, all patients displaying mutated *TP53* and *BIRC3* are in the CLL low group, as well as 5/6 patients with mutated *SF3B1*.

### 3.3. Response to Treatment with Ibrutinib

**Figure 3:**
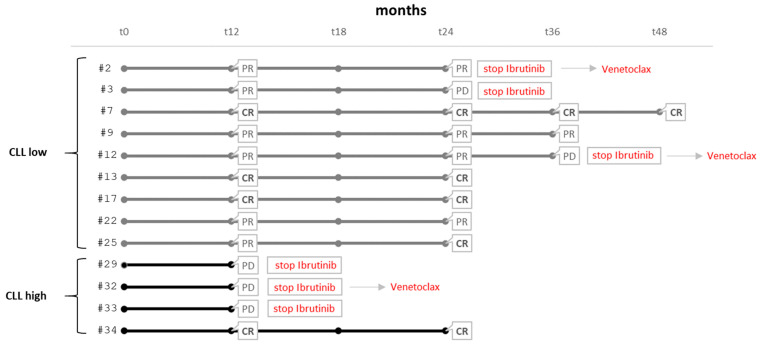
Responses to ibrutinib treatment in individual CLL high and low patients (in bold lines, CLL high; PD, progressive disease; CR, complete remission; PR, partial remission).

In total, 13 of the 35 patients underwent ibrutinib treatment according to clinical criteria and biological parameters. Thus, we decided to analyze whether the response to the BTK inhibitor could be associated with a different bioenergetic metabolism profile by isolating bioenergetic data from the previous analysis and analyzing them in correlation with clinical characteristics (Table 3) and ibrutinib response (Figure 3) in this subpopulation. None of the 13 patients carried *BTK* and *PLCG2* mutations, which are consistently associated with ibrutinib resistance [28].

Despite the small number of cases, the subdivision of patients into two clusters according to the basal bioenergetic metabolism (CLL low and CLL high) was preserved. In particular, 9 out of 13 patients showed a bioenergetic metabolic phenotype characterized by low OCR levels (mean value 26.57 ± 11.80 pmoles O_2_/min) and low ECAR levels (mean value 8.14 ± 4.15 mpH/min), while the remaining four patients presented a distinct bioenergetic metabolic phenotype, which is significantly higher compared to the low bioenergetic metabolism cluster in terms of both OCR (mean value 62.27 ± 14.15 pmoles O_2_/min, *p* < 0.0001 vs. low) and ECAR (mean value 20.79 ± 3.45 mpH/min, *p* < 0.0001 vs. low). These data were confirmed in the Mito Stress Test parameters (Supplemental Table S2).

The two patient clusters were once again analyzed based on the following clinical characteristics: sex, age, beta-2 microglobulin, LDH, WBC count, Rai and Binet staging, time from diagnosis, and previous therapies performed.

Similar to the overall population, a higher WBC count was observed in the CLL low group compared to the CLL high (median CLL low: 47,200—range 20,900–159,000; median CLL high: 17,955—range 3880–30,000), although in this subpopulation, the differences did not reach statistical significance (*p* = 0.07) due to the small sample size (Table 3). No correlations were found for the other parameters. A sample in the CLL high group was found to be inadequate for cytogenetic and molecular biology evaluation, and therefore it was not considered in the analysis of these results. As for the *IGHV* genes’ mutational status (Table 4), seven out of nine patients of the CLL low cluster had unmutated *IGHV* (*p* = 0.034). Except for a case of trisomy of chromosome 12, all of the analyzed cytogenetic and molecular alterations were detected in samples from patients belonging to the CLL low group, as summarized in Table 4. Each patient was evaluated for their response to ibrutinib, as reported in Figure 3.

From the analysis of the bioenergetic metabolic profile in relation to the response to ibrutinib, some differences emerged between the two clusters.

As shown in Figure 3, CLL low showed a better and longer response to treatment with ibrutinib. After 12 months of treatment, it was observed that in the CLL low cluster, 3/9 patients achieved CR and 6/9 obtained PR. In contrast, only one patient in the CLL high achieved CR, while three of four patients manifested PD and therefore discontinued treatment.

After 24 months follow-up (Figure 3), 4/9 CLL low patients were in CR, 4/9 maintained a PR, and only one patient exhibited PD. The only CLL high patient in CR maintained the response at 24 months.

## 4. Discussion

Aberrant cell metabolism is currently being recognized as a paramount factor in the response of cancer cells to therapeutic agents. Indeed, metabolic plasticity has been correlated to the resistance to chemotherapy [29]. An in-depth understanding of how cancer cells undertake a path of metabolic reprogramming with the acquisition of new bioenergetic phenotypes is useful to expand the current therapeutic strategies, avoiding the insurgence of resistance phenomena and thus improving patients’ prognosis.

In this regard, many efforts have been made to clarify the metabolic characteristics of the many tumor histotypes, as we can observe a heterogeneity in metabolism remodeling that reflects the dramatically different genetic and proteomic backgrounds of various tumors. CLL cells, as mentioned, differ from the majority of cancer cells, as they do not appear to be characterized by the Warburg effect, showing a mainly oxidative phenotype [30,31]. Glutamine and fatty acids fuel this oxidative phenotype, feeding the tricarboxylic acid cycle activity [13]. In the attempt to confer a prognostic significance to CLL cell metabolism, Vangapandu et al. [32] measured ECAR and OCR in samples from CLL patients compared to normal B cells and peripheral blood mononuclear cells (PBMC) from healthy donors, revealing differences between CLL samples. These differences reflected the aggressiveness of the disease, since a higher respiration correlated with unfavorable prognostic markers (higher Rai score, β2 microglobulin, Zap70, unmutated *IGHV*), while ECAR was quite similar among CLL samples [32]. Accordingly, our data show a variability in OCR rates between CLL cells from different patients, indicating dissimilar respiration rates that allows for the clustering of the investigated patient population into two groups according to a higher or lower O_2_ consumption. The lower OCR subgroup was no different to normal B cells, according to clustering analysis. Further dissection of mitochondrial metabolism by means of parameter analysis from Mito Stress Test experiments, such as basal, maximal, spare capacity, and ATP-linked respiration, all correlated with the subdivision in CLL low and CLL high. However, these differences in OCR were also accompanied by a variability in ECAR, suggesting a more active bioenergetic metabolism *in toto* in the CLL high subgroup. Interestingly, we found that CLL cells with a low bioenergetic metabolism differed from their normal B counterpart, with ECAR being higher in the latter.

Moreover, according to our data, CLL cells showing lower bioenergetic metabolic rates belonged to patients characterized by unfavorable molecular genetic factors. CLL low was in fact associated with a higher WBC, del(11q), and multiple deletions, as well as mutations in *TP53*, *BIRC3*, and *SF3B1*. On the other hand, three out of four cases with trisomy 12 were in the CLL high subgroup. Notably, these results are in contrast with those obtained from Vangapandu’s group [32]. However, there is a fundamental difference in the approach followed to perform experiments, as they incubated CLL cells in culture medium for 24h before metabolic parameter assessments. Our approach consisted in the rapid measurements of CLL cells’ bioenergetic metabolism right after the purification passage, with a brief (30 min) period of adaptation in culture medium to stabilize temperature, in the attempt to minimize the ex vivo artefacts. Additional samples are required to further refine these observations, and future analyses are planned to expand the results. In particular, the study of the proteomic profile could identify differential protein expression profiles in the two metabolic groups, thus revealing a specific target useful for reverting the metabolic phenotypes. Moreover, data from the literature shows the impact of the stromal microenvironment on CLL cell metabolism, but results are unclear if it is in favor of glycolysis [33] or OXPHOS [32].

Several studies have highlighted that the aberrant signaling caused by the constitutive activation of the BCR has a profound impact on the metabolism of CLL cells characterized by an increase in oxidative phosphorylation compared to its healthy counterpart [32]. Therefore, studying the metabolism in relation to the response to BCR signaling inhibitors represents a further element in the characterization of this disease. In fact, we intended to evaluate whether the presence of a peculiar metabolic phenotype can be predictive of the response to a specific BCR signaling inhibitor treatment. In total, 13 of the 35 patients enrolled in our study underwent ibrutinib treatment, 4 of which were treatment-naïve, while 9 had been previously treated with regimens based on chemo- or chemoimmunotherapy. Bioenergetic metabolic characterization was performed before starting ibrutinib, confirming a clear clustering of patients into two groups with low and high bioenergetic metabolism. The two groups showed a statistically significant difference both in terms of baseline OCR and Mito Stress Test parameters. Starting from this subdivision, an attempt was made to further investigate the difference observed in terms of oxygen consumption, comparing the data of mitochondrial metabolism with the major prognostic factors and with the response to ibrutinib treatment. According to our reduced sample population, no correlation was found with sex, age, beta-2 microglobulin, LDH, staging according to Rai and Binet, time from diagnosis and the start of ibrutinib, and number of previous therapies performed. However, analysis confirmed that the CLL low group was characterized by a markedly higher white blood cell count compared to the CLL high group. The most relevant unfavorable prognostic factors such as *TP53* mutation, del(13q) deletion, multiple deletions, *BIRC3* mutation, *SF3B1*, and *NOTCH1* mutation in association with trisomy 12 were present in the CLL low group. Interestingly, seven out of nine patients in the CLL low cluster had unmutated *IGHV* genes, which confers an increased sensitivity to ibrutinib, as reported in the literature.

As for our study, a better and more durable response to ibrutinib was observed in CLL low patients, 7/9 of whom remained on BTK inhibitor treatment for more than 24 months.

In a study by Guo et al. [34], the biological differences between mutated and unmutated *IGHV* CLL were analyzed: the results obtained revealed that the levels of BTK phosphorylation were significantly higher in the unmutated *IGHV* CLL group. In addition, the unmutated *IGHV* group presented an increased BCR signaling and a greater sensitivity to ibrutinib compared to mutated *IGHV* CLL [34]. We can therefore postulate that in the CLL low group, mostly with unmutated *IGHV*, the good response to ibrutinib is attributable to the cell shut down secondary to the deactivation of the BTK signaling, suggesting that these cells are addicted to this signaling for their survival. Notably, the only CLL high that reached and maintained CR for 24 months has unmutated *IGHV*. Although it has been reported in the literature that the response to ibrutinib is independent to the mutational status of *IGHV* [35], our data seem to indicate that their evaluation, in combination with other parameters such as the bioenergetic metabolic profile, can stratify patients for a different response to the inhibitor by BTK.

Moreover, the study of Lu et al. (Haematologica 2019), by using a different approach focusing on glycolysis, further highlights that heterogeneity in the CLL cells’ bioenergetic metabolism is influenced by genetic/molecular characteristics [36]. Lu et al., analyzing the capacity of the glycolytic pathway, observed an increased glycolytic activity in unmutated CLL as compared to mutated CLL, sustained by an upregulation of the glycolytic key enzymes [36]. The data shown by the authors suggest that in particular, glycolytic capacity and reserve parameters correlate with *IGHV* status and have a strong predictive value for overall survival [36]. Moreover, these parameters were linked to an increased resistance towards drugs that affect mitochondria (i.e., rotenone, venetoclax, and orlistat) [36].

Furthermore, two of the four CLL low patients who achieved a partial response had del(17p), one of which had it in association with *TP53* mutation. This underlines that, although the ibrutinib response seemed independent of clinical and genomic risk factors, del(17p) and *TP53* aberrations could remain unfavorable prognostic factors when using the BTK inhibitor as continuous monotherapy [3,37,38]. Several studies have already reported the effect of *TP53* mutations on the regulation of cancer metabolism, although, to date, the role of this alteration on CLL metabolism has not been fully elucidated. Eriksson et al. [39], by determining ECAR and OCR in various types of tumor cells expressing different *TP53* mutations, showed that even the same amino acid substitutions in the p53 protein can have extremely different phenotypic effects in terms of metabolism, depending on the origin of the cell line. In particular, the authors demonstrated that, while the glycolytic changes induced by *TP53* mutations can be kept consistent in cancer cells, the pathways of mitochondrial energy metabolism are influenced by the type of *TP53* mutant: some cause an increase, and others cause a decrease [39]. Looking at our data, CLL could fall into this second group, and thus *TP53* could represent a further element of susceptibility to therapies, and in particular to BTK inhibitors.

In contrast, in CLL high patients, the BTK signaling may not represent the main regulator of cell survival, and therefore the use of ibrutinib may not prove to be the most effective target therapy, at least as a single treatment. Therefore, a useful alternative approach could be the inhibition of other pathways, also in combination with ibrutinib. Vangapandu et al. [32], focusing on mitochondrial metabolism, in fact demonstrated how the pharmacological inhibition of PI3K (by duvelisib and idelalisib) caused a decrease in cellular OCR levels.

In addition to the *IGHV* mutational status, trisomy 12 has been reported to be a determinant of ibrutinib sensitivity. In our study group, the two patients who obtained a better and more durable response to ibrutinib both had unmutated-IGHV and trisomy 12, but displayed a divergent bioenergetic metabolic profile (being one of the CLL low group and one of the CLL high group). This could suggest that the association of these two factors strongly determines the sensitivity to BTK inhibitor, regardless of the metabolic profile. Whether we are aware that the main limitation of our study is the small sample size for the patient undergoing ibrutinib treatment, this observation deserves, in our opinion, to be further investigated in a larger number of patients in searching for potential predictive factors of ibrutinib response.

In conclusion, we have shown that, after analyzing the levels of ECAR and OCR, CLL patients displayed two different bioenergetic metabolic profiles, and that these are associated with biological characteristics: CLL cells displaying low bioenergetic metabolism are characterized, in fact, by a higher WBC count and unfavorable prognostic factors. The two bioenergetic metabolic profiles also seem to indicate different efficacy of the targeted therapy with ibrutinib, with the group characterized by lower bioenergetic metabolism showing a more durable response, thus finding a potential patients’ subgroup that can benefit the most from this therapy regimen.

## Figures and Tables

**Table 1:** Clinical and biological parameters associated with the OCR and ECAR values obtained using the bioenergetic metabolic analysis for each patient (neg: negative; nd: not determined; OCR is expressed as pmolesO_2_/min, ECAR is expressed as mpH/min).

pt n.	WBC	*IgHV*	MUTATIONS	CYTOGENETICS	OCR Mean	OCR SD	ECAR Mean	ECAR SD
low								
#1	37210	unmutated		Del13q14.3; del11q22.3	17.90	0.64	2.36	0.98
#2	73290	unmutated		del13q14.3; del 17p (15%)	23.01	5.53	6.56	1.04
#3	159000	unmutated	*SF3B1*	neg	10.48	1.75	2.39	0.25
#4	32500	mutated		del13	8.67	1.34	2.10	0.26
#5	27900	mutated		del 13q	17.20	2.81	6.92	0.38
#6	50000	mutated		del 13q14.3	16.75	3.58	7.16	0.30
#7	25000	unmutated	*NOTCH1*	tris 12	40.87	3.34	10.57	1.16
#8	80600	unmutated		del11q22.3	7.06	2.12	1.54	0.05
#9	20900	unmutated		neg	44.49	2.21	12.53	1.30
#10	49200	mutated		neg	31.45	4.18	11.59	1.89
#11	140000	unmutated		del11	19.43	6.33	6.58	2.56
#12	102400	unmutated	*TP53*, *SF3B1*	del 17p; del 11q	23.86	2.06	10.98	0.54
#13	47200	mutated	*SF3B1*	del 13q14.3; del11q22.3	19.79	1.45	5.75	0.32
#14	79230	mutated		neg	27.43	4.12	8.44	1.95
#15	77240	mutated		del13	4.50	3.56	2.40	0.82
#16	40940	mutated		del13	21.74	2.44	6.08	0.58
#17	40400	mutated		del 13q14.3	23.46	8.73	7.43	1.91
#18	25400	mutated		del13	17.71	2.11	5.95	0.96
#19	76200	mutated		neg	11.57	1.37	4.47	0.92
#20	16300	unmutated	*TP53*	del 13q14.3	32.83	1.23	12.53	0.16
#21	41600	unmutated	*NOTCH1*	del 13q14.3 (7%)	36.21	2.40	14.01	0.65
#22	78000	unmutated	*NOTCH1*	del13	15.95	4.90	2.72	0.62
#23	92300	unmutated	*TP53*, *SF3B1*	del17;tris12;del13	27.73	6.42	6.71	1.99
#24	15300	mutated	*SF3B1*	neg	15.72	2.62	5.72	0.14
#25	26800	unmutated		neg	37.25	4.70	14.32	1.08
#26	50000	mutated	*TP53*	del17:del13	34.21	2.50	13.38	0.94
high								
#27	15100	unmutated		tris 12	51.88	1.10	21.07	3.13
#28	4600	mutated	*SF3B1*	neg	56.42	9.17	24.86	4.14
#29	30000	mutated		nd	75.60	3.46	20.29	0.81
#30	32290	unmutated		tris 12	68.23	1.49	22.83	1.59
#31	15400	mutated		del 13q14.3	71.15	6.37	21.30	1.81
#32	3880			nd	46.02	19.65	20.56	7.68
#33	21100	mutated		neg	66.63	1.96	20.18	0.37
#34	14810	unmutated		tris 12	60.83	2.07	22.14	1.43
#35	6040	mutated		nd	69.08	10.05	22.06	4.93

**Table 2:** Cytogenetic and molecular characteristics of patients according to bioenergetic metabolic status (n.s. = not significative).

	Tot	CLL High (%tot)	CLL Low (%tot)	*p*
***IGHV* Mutated**	18	5 (27.8%)	13 (72.2%)	n.s.
***IGHV* Unmutated**	16	3 (18.8%)	13 (81.2%)	0.0124
**del(13q)**	11	1 (9.1%)	10 (90.9%)	0.0067
**del(17p)**	0	0 (0%)	0 (0%)	n.s.
**del(11q)**	2	0 (0%)	2 (100%)	n.s.
**del(13q);del(17p)**	3	0 (0%)	3 (100%)	n.s.
**del(11q);del(17p)**	1	0 (0%)	1 (100%)	n.s.
**del(11q);del(13q)**	3	0 (0%)	3 (100%)	n.s.
**Tris12**	4	1 (25%)	3 (75%)	n.s.
***TP53***	4	0 (0%)	4 (100%)	0.046
***NOTCH1***	2	0 (0%)	2 (100%)	n.s.
***BIRC3***	5	0 (0%)	5 (100%)	0.025
***SF3B1***	6	1 (16.7%)	5 (83.3%)	n.s.

**Table 3:** Clinical and biological parameters associated with OCR and ECAR value obtained from bioenergetic metabolic analysis for each patient receiving ibrutinib treatment (neg: negative; nd: not determined; OCR is expressed as pmolesO_2_/min, ECAR is expressed as mpH/min).

pt n.	WBC	*IgHV*	MUTATIONS	CYTOGENETICS	OCR Mean	OCR SD	ECAR Mean	ECAR SD
low								
#2	73290	unmutated		del13q14.3; del 17p (15%)	23.01	5.53	6.56	1.04
#3	159000	unmutated	*SF3B1*	neg	10.48	1.75	2.39	0.25
#7	25000	unmutated	*NOTCH1*	tris 12	40.87	3.34	10.57	1.16
#9	20900	unmutated		neg	44.49	2.21	12.53	1.3
#12	102400	unmutated	*TP53*, *SF3B1*	del 17p; del 11q	23.86	2.06	10.98	0.54
#13	47200	mutated	*SF3B1*	del 13q14.3; del11q22.3	19.79	1.45	5.75	0.32
#17	40400	mutated		del 13q14.3	23.46	8.73	7.43	1.91
#22	78000	unmutated	*NOTCH1*	del13	15.95	4.9	2.72	0.62
#25	26800	unmutated		neg	37.25	4.7	14.32	1.08
high								
#29	30000	mutated		nd	75.6	3.46	20.29	0.81
#32	3880			nv	46.02	19.65	20.56	7.68
#33	21100	mutated		no	66.63	1.96	20.18	0.37
#34	14810	unmutated		tris 12	60.83	2.07	22.14	1.43

**Table 4:** Cytogenetic and molecular characteristics of patients undergoing ibrutinib treatment according to their bioenergetic metabolic status.

	Tot (12)	CLL High (3—%tot)	CLL Low (9—%tot)
***IGHV* MUTATED**	4	2 (50%)	2 (50%)
***IGHV* UNMUTATED**	8	1 (12.5%)	7 (87.5%)
**del(13q)**	1	0 (0%)	1 (100%)
**del(17q)**	0	0 (0%)	0 (0%)
**del(11p)**	0	0 (0%)	0 (0%)
**del(13q);del(17p)**	1	0 (0%)	1 (100%)
**del(11q);del(17p)**	1	0 (0%)	1 (100%)
**del(11q);del(13q)**	1	0 (0%)	1 (100%)
**Tris12**	2	1 (50%)	1 (50%)
***TP53***	1	0 (0%)	1 (100%)
***NOTCH1***	1	0 (0%)	1 (100%)
***BIRC3***	3	0 (0%)	3 (100%)
***SF3B1***	3	0 (0%)	3 (100%)

## Data Availability

The original contributions presented in the study are included in the article/supplementary materials, further inquiries can be directed to the corresponding author/s.

## Supplementary Materials

The following supporting information can be downloaded at: https://www.mdpi.com/article/10.3390/cimb46060305/s1, Table S1: previous lines of therapy for each patient (FCR: Fludarabine-cyclophosphamide-rituximab; R CVP: rituximab plus cyclophosphamide, vincristine, and prednisone); Table S2: Mitochondrial parameters obtained from mito stress test for each CLL low and CLL high patient. Results are expressed in pmolesO_2_/min.
