# Supplementary material for: Low Cell Bioenergetic Metabolism Characterizes Chronic Lymphocytic Leukemia Patients with Unfavorable Genetic Factors and with a Better Response to BTK Inhibition
Source: Curr Issues Mol Biol. 2024 May 22;46(6):5085–99. doi: 10.3390/cimb46060305 (PMC11202558; doi:10.3390/cimb46060305)
Supplement: Supplementary file 1 [file cimb-46-00305-s001.zip › cimb-2951538-supplementary.pdf]

Supplementary Table 1: previous lines of therapy for each patient (FCR: Fludarabine-cyclophosphamide-rituximab; R CVP: rituximab plus cyclophosphamide, vincristine, and prednisone)

PT N.	AGE	SEX	RAI	BINET	WBC	LDH	BETA2MG	CD38	CD49D	IGHV	MUTATIONS	CYTOGENETICS	I LINE	II LINE	III LINE
LOW															
#1	66	M	3	C	37210	419	1.99	neg	neg	unmutated		Del13q14.3; del11q22.3	FCR		
#2	76	F	1	B	73290	180		neg	neg	unmutated		del13q14.3; del 17 q (15%)	R CHLORAMBUCIL	Imbruvica	
#3	81	M	4	C	159000			neg	neg	unmutated	SF3B1	neg	LEUKERAN	Fludarabine	R CVP
#4	59	M	2	B	32500	134		neg	neg	mutated		del13	NO PREVIOUS TREATMENT		
#5	74	M	1	B	27900	388	1.8	neg	neg	mutated		del 13q	NO PREVIOUS TREATMENT		
#6	71	F	3	C	50000	162	2.15	neg	neg	mutated		del 13q14.3	NO PREVIOUS TREATMENT		
#07	83	M	2	B	25000	397	2.69	neg	neg	unmutated	NOTCH1	tris 12	R CHLORAMBUCIL	R Bendamustina	Imbruvica
#8	81	F	4	C	80600	343		neg	neg	unmutated		del11q22.3	NO PREVIOUS TREATMENT		
#9	86	M	4	C	20900	202		neg	neg	unmutated		neg	R LEUKERAN	Imbruvica	
#10	64	M	2	B	49200	201		neg	neg	mutated		neg	NO PREVIOUS TREATMENT		
#11	78	F	2	B	140000	148		neg	neg	unmutated		del11	LEUKERAN		
#12	74	M	2	B	102400	162		19%	6%	unmutated	TP53, SF3B1	del 17p; del 11q	FCR	Imbruvica	
#13	81	F	2	B	47200	206		neg	neg	mutated	SF3B1	del 13q14.3; del11q22.3	NO PREVIOUS TREATMENT		
#14	78	M	2	A	79230	473		neg	neg	mutated		neg	NO PREVIOUS TREATMENT		
#15	61	M	2	B	77240	150	2,21	neg	neg	mutated		del13	NO PREVIOUS TREATMENT		
#16	77	F	2	B	40940	144	2.72	neg	neg	mutated		del13	NO PREVIOUS TREATMENT		
#17	86	M	2	B	40400	178		neg	neg	mutated		del 13q14.3	CHLORAMBUCIL		
#18	66	F	1	B	25400	171	1.7	neg	neg	mutated		del13	NO PREVIOUS TREATMENT		
#19	78	F	2	B	76200	336	2.5	neg	neg	mutated		neg	NO PREVIOUS TREATMENT		
#20	83	F	2	B	16300	225	0.2	0,76	neg	unmutated	TP53	del 13q14.3	R CHLORAMBUCIL	R Chlorambucil	Imbruvica
#21	58	M	1	B	41600	416	1.6	neg	neg	unmutated	NOTCH1	del 13q14.3 (7%)	NO PREVIOUS TREATMENT		
#22	85	F			78000	286		0,28	nv	unmutated	NOTCH1	del13	IBRUTINIB		
#23	80	F			92300	620		0,07	nv	unmutated	TP53, SF3B1	del17;tris12;del13	CHLORAMBUCIL	gazyvaro	imbruvica
#24	52	M			15300	220		<7%	<7%	mutated	SF3B1	neg	NO PREVIOUS TREATMENT		
#25	73	M			26800	194		neg	neg	unmutated		neg	IBRUTINIB		
#26	85	M			50000	195		5%	nv	mutated	TP53	del17:del13	IBRUTINIB		
HIGH															
#27	79	F	4	C	15100	269	2.82	neg	neg	unmutated		tris 12	NO PREVIOUS TREATMENT		
#28	84	F	2	B	4600	435		neg	neg	mutated	SF3B1	neg	R CHLORAMBUCIL		
#29	86	M	2	B	30000	154		6%	neg	mutated		nv	R CHLORAMBUCIL	Imbruvica	
#30	86	F	3	C	32290	211	1,9	56%	neg	unmutated		tris 12	LEUKERAN		
#31	51	M	0	A	15400	206	2.19	neg	neg	mutated		del 13q14.3	NO PREVIOUS TREATMENT		
#32	79	M	2	B	3880	285	2.0	neg	pos			nd	FCR	Imbruvica	Venetoclax
#33	84	M	3	C	21100	475	3.12	neg	neg	mutated		neg	IMBRUVICA		
#34	75	M	2	B	14810	214		neg	neg	unmutated		tris 12	R BENDAMUSTINE	Imbruvica	

**#35**	71	F	6040	21<br>1	mutated	nd	NO PREVIOUS TREATMENT

Supplementary Table 2. Mitochondrial parameters obtained from mito stress test (fig.1a) for each CLL low and CLL high patient. Results are expressed in pmolesO2/min

	**BASAL RESPIRATION**		**MAXIMAL RESPIRATION**		**ATP PRODUCTION**		**SPARE RESPIRATORY CAPACITY**		**SPC AS %**		**COUPLING EFFICIENCY**	
**PT N.**	mean	SD	mean	SD	mean	SD	mean	SD	mean	SD	mean	SD
LOW												
**#1**	7.59	1.73	18.47	1.02	9.25	0.68	10.88	0.77	2.49	0.39	1.24	0.17
**#2**	14.59	2.72	28.12	7.67	16.15	2.53	13.52	4.97	1.90	0.19	1.11	0.05
**#3**	6.59	1.78	7.32	0.92	7.33	1.19	0.73	2.39	1.19	0.48	1.14	0.15
**#4**	5.60	1.42	10.83	1.40	4.90	1.90	4.90	1.90	1.98	0.31	0.89	0.35
**#5**	20.53	10.94	15.89	2.48	15.61	5.15	-4.63	9.84	1.02	0.71	0.88	0.33
**#6**	21.26	4.73	22.50	3.53	15.73	1.87	1.24	1.39	1.07	0.09	0.78	0.28
**#7**	19.25	1.96	61.57	3.84	24.31	2.39	42.31	1.88	3.21	0.12	1.26	0.06
**#8**	2.04	0.79	6.53	0.53	3.32	0.78	4.49	0.52	3.60	1.56	1.72	0.38
**#9**	28.87	1.39	66.32	11.03	28.31	1.10	37.46	10.06	2.29	0.30	0.98	0.04
**#10**	17.48	2.34	17.63	6.60	15.98	5.65	0.14	4.45	0.99	0.25	0.90	0.25
**#11**	13.00	5.73	7.37	2.87	12.02	5.23	-5.63	4.15	0.61	0.25	0.94	0.12
**#12**	14.18	1.77	24.15	2.47	9.71	0.60	9.96	0.77	1.71	0.05	0.69	0.11
**#13**	14.25	1.31	15.30	0.51	11.26	1.63	1.05	1.62	1.08	0.12	0.79	0.04
**#14**	18.95	3.88	22.00	7.96	15.47	1.05	3.06	4.79	1.14	0.21	0.83	0.12
**#15**	4.34	1.23	10.76	0.72	5.08	2.07	6.42	0.51	2.57	0.49	1.28	0.75
**#18**	12.18	0.87	12.22	2.74	12.51	1.44	0.03	2.05	1.00	0.17	1.02	0.06
**#19**	8.85	0.83	9.22	0.56	7.93	0.81	0.37	0.80	1.05	0.09	0.90	0.02
**#20**	19.58	1.46	91.75	3.21	26.20	0.86	72.18	1.95	4.70	0.20	1.34	0.05
**#21**	20.61	0.13	81.62	8.38	21.20	1.99	61.01	8.51	3.96	0.43	1.03	0.10
**#22**	8.38	1.53	4.67	2.07	7.06	1.27	-3.71	2.21	0.56	0.27	0.84	0.05
**#23**	16.36	4.40	20.89	6.98	7.13	5.00	4.52	3.86	1.28	0.24	0.47	0.35
**#24**	8.96	0.85	15.75	5.89	5.85	5.01	6.78	5.05	1.73	0.47	0.69	0.60
**25**	22.75	2.72	23.20	5.05	22.76	0.81	0.46	2.45	1.01	0.10	1.01	0.12
**#26**	20.55	2.70	34.47	2.96	20.55	0.93	13.92	1.30	1.69	0.12	1.01	0.14
HIGH												
**#27**	35.43	1.68	178.22	3.49	33.89	1.74	142.78	4.63	5.04	0.30	0.96	0.01
**#28**	36.80	4.22	162.81	33.99	34.28	4.05	126.01	29.80	4.39	0.45	0.93	0.00
**#29**	35.77	2.85	164.86	2.59	30.34	1.60	129.09	4.64	4.63	0.40	0.85	0.04
**#30**	42.45	1.39	158.25	45.73	46.69	1.49	115.81	47.10	3.75	1.19	1.10	0.07
**#31**	40.01	11.77	233.76	60.97	45.36	6.54	193.75	50.54	5.92	0.67	1.18	0.26
**#32**	33.21	12.37	97.44	70.64	32.92	12.76	64.23	59.00	2.70	1.04	0.99	0.03
**#33**	41.81	4.74	248.94	10.24	47.00	1.82	207.13	7.44	5.99	0.51	1.13	0.09
**#34**	41.01	1.27	173.08	26.20	43.66	1.25	132.08	25.72	4.22	0.61	1.07	0.04
**#35**	44.53	6.89	111.02	37.70	44.03	6.02	66.48	35.60	2.50	0.85	0.61	0.56
B NORM												
**#B1**	3.42	1.77	7.97	1.68	4.76	1.57	4.55	0.24	2.59	0.81	1.53	0.57
**#B2**	19.49	4.06	34.03	5.93	17.74	3.07	14.54	2.57	1.76	0.13	0.92	0.07
**#B3**	3.46	4.32	13.13	1.71	6.63	1.04	9.67	2.65	-1.71	7.69	-1.61	4.81
**#B4**	26.93	3.49	64.31	20.95	25.36	3.24	37.38	18.26	2.36	0.59	0.94	0.05

**#B5**	21.27	0.26	35.23	4.88	20.33	1.34	13.96	4.83	1.66	0.23	0.96	0.06
**B6**	24.24	5.61	49.40	13.65	25.21	5.95	25.15	8.68	2.03	0.20	1.04	0.11
